# A Facile Method for Batch Preparation of Electrochemically Reduced Graphene Oxide

**DOI:** 10.3390/nano9030376

**Published:** 2019-03-05

**Authors:** Yi-Fang Hung, Chia Cheng, Chun-Kai Huang, Chii-Rong Yang

**Affiliations:** Department of Mechatronic Engineering, National Taiwan Normal University, Taipei 106, Taiwan; yfhongiou@gmail.com (Y.-F.H.); giles540624@gmail.com (C.C.); roy85201@hotmail.com (C.-K.H.)

**Keywords:** electrochemically reduced graphene oxide, graphene oxide, phosphate buffered saline, Raman spectroscopy, X-ray photoelectron spectroscopy

## Abstract

The electrochemical reduction of graphene oxide (GO) is an environmentally friendly and energy-saving method for improving the characteristics of GO. However, GO films must be coated on the cathode electrode in advance when usingthis technique. Thus, the formed electrochemically reduced GO (ERGO) films can be used only as sensing or conductive materials in devices because mass production of the flakes is not possible. Therefore, this study proposes a facile electrochemical reduction technique. In this technique, GO flakes can be directly used as reduced materials, and no GO films are required in advance. A 0.1 M phosphate buffered saline solution was used as the electrolyte, which is a highly safe chemical agent. Experimental results revealed that the as-prepared GO flakes were electrochemically reduced to form ERGO flakes by using a −10 V bias for 8 h. The ratio of the D-band and G-band feature peaks was increased from 0.86 to 1.12, as revealed by Raman spectroscopy, the π-π stacking interaction operating between the ERGO and GO has been revealed by UV-Vis absorption spectroscopy, and the C–O ratio was increased from 2.02 to 2.56, as indicated by X-ray photoelectron spectroscopy. The electrical conductivity of the ERGO film (3.83 × 10^−1^ S·cm^−1^) was considerably better than that of the GO film (7.92 × 10^−4^ S·cm^−1^). These results demonstrate that the proposed electrochemical reduction technique can provide high-quality ERGO flakes and that it has potential for large-scale production.

## 1. Introduction

Graphene is a two-dimensional material with a hexagonal honeycomb crystal lattice in which carbon atoms consist of sp^2^ hybrid orbitals; graphene was discovered by Geim and Novoselov in 2004, who received the 2010 Nobel Prize in physics [1]. This material has been attracting considerable scientific and technological interest because it has unique physical and chemical properties [2,3,4], such as a large specific surface area, excellent electrical conductivity and thermal conductivity, high mechanical strength, high electron mobility, and visible transparency. Therefore, graphene has considerable potential applications in many fields, such as gas sensors, biosensors [5], ultra-high-speed transistors, electronic and optoelectric devices [6,7,8], solar cells [9], supercapacitors [10], high-efficiency lithium batteries [11], and energy storage devices [12].

Currently, GO and reduced GO (RGO) are trending topics in the research and development of graphene [13,14,15,16], especially concerning large-scale applications. GO and RGO production processes are easily scalable and can be used for mass production using inexpensive graphite as a raw material, thus yielding cost-effective chemical methods with a high yield. In addition, GO–RGO materials have a high specific surface area and covalent oxygen functional groups; thus, they can be easily modified chemically and used in energy storage [17,18] and biosensing [19] applications.

The chemical exfoliation of graphite through oxidation leads to covalent functionalization of oxygen-containing groups, which dramatically alters the structure and property of graphene and causes GO to act as an electrical insulator and to have poor thermal conductivity. Therefore, the reduction of GO is clearly a key subject. Different reduction processes result in distinct properties that in turn affect the final performance of materials or devices comprising RGO. Scientists have attempted to heal GO to graphene through methods such as chemically reduced GO (CRGO) using hydrazine (N_2_H_4_) [20] or sodium borohydride (NaBH_4_) [21], thermally reduced GO (TRGO) in an argon atmosphere at approximately 1000 °C [22], plasma treatment [23], laser treatment [24], microwave treatment [25], photocatalytic treatment [26], aluminum powder [27], vitamin C [28], orsodium citrate [29]. The ultimate goal in all cases is to restructure the characteristic graphitic sp^2^ network.However, these GO reduction methods require poisonous chemical agents, considerable energy consumption, special equipment, or anunsatisfied characteristic of RGO. Thus, these methods require further improvement.

Compared with the aforementioned methods, electrochemical reduction is an environmentally friendly and energy-saving method. In this method, a reaction is conducted in an electrolyte using a bias voltage generated through a power supply system, and a three-electrode system with a working electrode on which GO films were deposited is generally used to apply a negative voltage to produce RGO through a reductive reaction [30]. However, to date, electrochemical reduction has typically been conducted through cyclic voltammetry or a constant potential mode by using a working electrode with deposited GO films [31,32,33]. Due to the electrochemical reaction between the working and counter electrodes, a change in appearance is obviously observed. That is, the brown color of GO is modified to black when RGO is formed. However, the GO films must be coated on the surface of the cathode in advance by using a drop-casting, dip-coating, spraying, spin-coating, electrophoresis, Langmuir–Blodgett, or rod-coating technique. In general, electrochemically RGO (ERGO) is directly used as a sensing or conductive material in sensing devices [34,35]. Nevertheless, electrochemical reduction cannot be used for the mass production of ERGO flakes; thus, the method requires further improvement.

This study proposes an electrochemical reduction technique that is suitable for batch production. Withthis technique, GO flakes can be directly used as reduced materials, and do not require the coating of GO films in advance. Furthermore, this technique directly reduces the GO flakes without the need to predry the GO film, which makes the run-to-run processes easy to complete. The proposed electrochemical reduction technique has potential for realizing large-scale and high-quality GO reduction.

## 2. Experimental Method

### 2.1. Graphene Oxide (GO)Preparation

GO was prepared using the modified Hummer’s method [36]. Natural graphite powder (200 mesh, Showa Kako) was the initial constituent. The graphite powder (3 g) was first incubated in H_2_SO_4_ (98%, 18 mL) with stirring and stored at 80 °C for 4.5 h. Subsequently, the solution was cooled down to room temperature and sonicated in a water bath for 2 h to obtain preoxidized graphite powder. The solution was then diluted with 0.75-L deionized (DI) water and left overnight. The preoxidized graphite powder was obtained after filtering the solution by using a vacuum filter. For further deep-level oxidation (using H_3_PO_4_ (40 mL) and H_2_SO_4_ (360 mL)) of the preoxidized graphite powder, the powder was added into H_3_PO_4_ (40 mL) and H_2_SO_4_ (360 mL), after which KMnO_4_ (18 g) was slowly added to the mixture under ice-bath cooling; the mixture was then stirred for 1 h. This deep-level oxidation process was conducted at a constant temperature of 50 °C for 12 h, and ice was used for rapid cooling. Subsequently, 15 mL of H_2_O_2_ (30%) was added to the mixture, and the mixture was placed stationary overnight. The color of the suspension changed from purple to yellowish brown. To remove the metal ions in the oxidized graphite powder, the powder was dissolved in HCl solution (HCl:H_2_O = 1:10 (volume ratio)) and recovered by centrifugation at 8500 rpm. The oxidized graphite powders were further dissolved repeatedly in DI water and recovered after centrifugationto remove the unwanted HCl until the pH of the solution was approximately equal to 7. To exfoliate the oxidized graphite slurry to form single-layer GO flakes, horn sonication was conducted for 30 min, and the GO slurry was recovered by conducting centrifugation. Finally, the as-synthesized GO slurry was dispersed into individual flakes in DI water to form a GO suspension of a concentration of 3 mg/mL through ultrasound for further use.

### 2.2. Electrochemically Reduced GO

**Figure 1:**
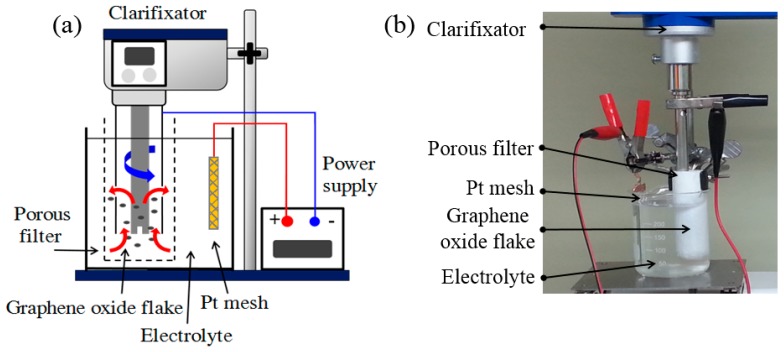
(**a**) Schematic of anexperiment setup, and (**b**) its actual photograph of electrochemical working area.

This study developed an apparatus—equipped with a DC power supply (GPC-6030D, GW, Taipei, Taiwan)—suitable for the electrochemical reduction of GO flakes (not GO film), as presented in Figure 1. A 0.1 M phosphate buffered saline (PBS) solution prepared using K_2_HPO_4_/KH_2_PO_4_ served as the electrolyte [37]. The PBS solution has a pH of 5.0 and is a safe and environmentally friendly chemical agent commonly used in biological research. A GO suspension (50 mL) was first poured into a porous glass filter cylinder with pore size of 1 μm (86R, Advantec, Tokyo, Japan) and then immersed into the PBS electrolyte (500 mL) in the beaker. A clarifixator (BOM-300D, Chemist, New Taipei, Taiwan) was used to cyclically stir the electrolyte and GO flakes for dispersing them in the porous glass filter cylinder using an assembled rotor–stator tool (HG-12, Chemist, New Taipei, Taiwan) fabricated from stainless steel 316L. A conventional two-electrode electrochemical cell was used. A rotor–stator tool was also used as the cathodic electrode, and a platinum-coated titanium mesh was used as the anodic electrode. The distance between the cathodic and anodic electrodes was approximately 2 cm. All experiments were conducted at ambient temperature.

When the electrochemical reduction experiments were conducted, the GO suspension in the porous ceramic filter cylinder was first fully agitated using the rotor–stator tool at 1000 rpm for 30 min to disperse the GO flakes. The bias voltage had yet to be applied at this stage; thus, only mechanical agitation was executed. When a bias voltage of −10 V was applied to the rotational tool, the electrochemical reduction process was initiated. A sample group produced using a bias of +10 V for 4 h was used as the control group. The reduction time was set to 2–8 h, and the agitation was maintained at 5000 rpm during the reduction experiment. After the electrochemical reduction of GO, the ERGO flakes were further dissolved repeatedly in DI water and recovered after centrifugation at 8500 rpm to remove the unwanted PBS solution until the pH was equal to approximately 7. The salts in the ERGO products obtained using electrochemical reduction through PBS could be easily removed through washing with water, thus minimizing the problem of waste liquid. Finally, the ERGO flakes were collected and dried in the shade before characterization.

### 2.3. Measurement and Characterization

The crystal structures of the as-prepared GO and ERGO samples were characterized using an X-ray diffraction (XRD) instrument (New D8 Discover, Bruker, Karlsruhe, Germany). Raman scattering is highly sensitive to electronic structures. Thus, Raman spectra were used to estimate the bonding characteristics; the spectra were measured under a green laser light with a wavelength of 532 nm (NRS-4100, Jasco, Tokyo, Japan).Ultraviolet-visible (UV-Vis) absorption measurement was carried out using a spectrophotometer (V670, Jasco, Tokyo, Japan).Scanning electron microscopy (SEM) was conducted to observe the sample surface (JSM-7610F, JEOL, Tokyo, Japan). Microstructure measurements were performed using transmission electron microscopy (TEM) with an accelerating voltage of 200 kV (JEM-2100F, JEOL, Tokyo, Japan). The sample for TEM characterization was prepared by placing a drop of suspension solution on a carbon-coated copper grid and drying the solution at room temperature. Atomic force microscopy (AFM) was used to examine the morphology and thickness of samples (Dimension Icon, Bruker, Santa Barbara, CA). X-ray photoelectron spectroscopy (XPS) was conducted to measure the surface chemistry properties of GO and ERGO. XPS was executed using a photoelectron spectrometer with Al as the excitation source (PHI-5000, ULVAC-PHI, Osaka, Japan). The thermal properties of the samples were characterized through thermogravimetric analysis (TGA) (Q500, TA, Leatherhead, UK). Fourier transform infrared (FTIR) spectra were used to record chemical bonds and functional groups by using a spectrophotometer (Vertex-80v, Bruker, Ettlingen, Germany). Sheet resistance and electrical conductivity were measured using a four-point probe resistance meter (5601Y, Chitai Electronic, Taipei, Taiwan).

## 3. Results and Discussion

**Figure 2:**
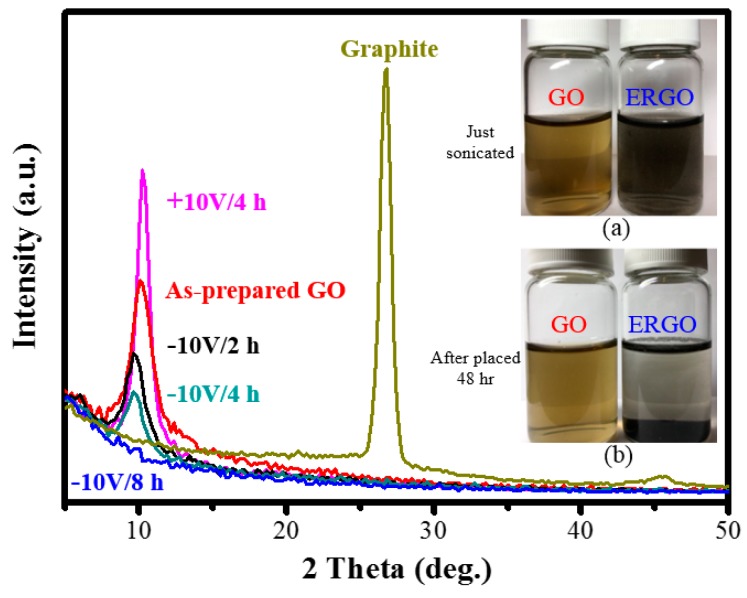
Comparison of XRD patterns among graphite, as-prepared GO and ERGO (+10 V/4 h, −10 V/2 h, −10 V/4 h, −10 V/8 h) samples. GO and ERGO (−10V/8h) suspensions (**a**) just sonicated and (**b**) after placed 48 h.

We first characterized the crystal structure of pristine graphite, the as-prepared GO (0 V), and ERGO (−10 V for 8 h, −10 V for 4 h, −10 V for 2 h, and +10 V for 4 h) samples, as presented in Figure 2. The feature diffraction peak of the as-prepared GO appeared at a 2θ value of 10.2° (002) with an interlay space (d-spacing) of 0.87 nm [38]. This d-spacing value was determined to be higher than that (0.34 nm) of pristine graphite (2θ = 26.5°) [39,40]. The reason for the higher d-spacing is that the van der Waals interactions between layers weakened due to of the formation of hydroxyl, epoxy, and carboxyl groups on carbon sheets, thus causing the water molecules to be intercalated between the layers. The interlayer distance of the ERGO products obtained after electrochemical treatment using a −10 V bias for 2, 4, and 8 h was expected to shrink due to the removal of the oxygenated functional groups [21]. However, the peak at 10.2° (0.87 nm) of the as-prepared GO gradually shifted to 9.9° (0.89 nm), 9.8° (0.90 nm), and no peak for the 2, 4, and 8h treatments, respectively. The interlay space of the ERGO flakes slightly increased. This increase indicates that K^+^ in the PBS electrolyte was incorporated into the ERGO structure and the ionic radius (152 Å) of K^+^ was larger than that (126 Å) of O^2−^ in the oxygenated functional groups, thus leading to a slight increase in the interlayer distance [41]. The reductive effect of ERGO flakes can be first judged visually from the color appearance. The yellowish brown GO solution turned to black, and a stable dispersion of ERGO flakes could be detected by the naked eye, as displayed in Figure 2a. The GO and ERGO suspensions had the same concentration of 0.3 g/mL and presented an obvious difference in color before and after the electrochemical reduction. As presented in Figure 2b, ERGO exhibited an obvious sedimentation process after being settled for 48 h, but GO was evenly dispersed. In general, graphene tends to aggregate and precipitate in aqueous media due to its hydrophobicity and the strongπ–π interaction between graphene layers [42]. These observations provide evidence to support the formation of ERGO [43,44].

**Figure 3:**
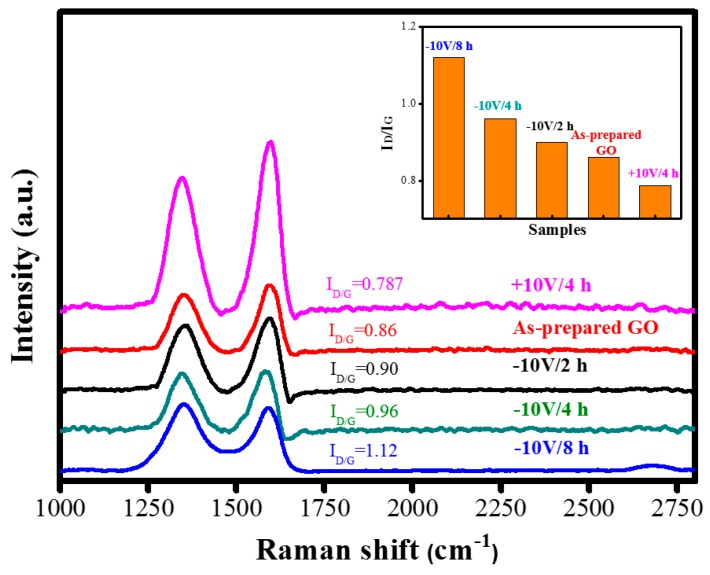
Comparison of Raman analysis among as-prepared GO and ERGO (+10 V/4 h, −10 V/2 h, −10 V/4 h, −10 V/8 h) samples. (Inset: Plot of I_D_/I_G_ of samples.)

The electrochemical reduction effect was further monitored using Raman spectra. Figure 3 presents a comparison of the graphene quality of the as-prepared GO and ERGO flakes. The Raman spectra of the as-prepared GO and ERGO flakes were prepared at different voltages (+10 V and −10 V). The Raman shift was measured in the range of 1000 to 2800 cm^−1^. A study [45] indicated that the grain boundary size of ERGO decreases after the reduction of the as-prepared GO; hence, the defect peak (D peak) of ERGO increases in size. The measured spectra demonstrated two characteristic peaks for the samples, namely the D band centered at approximately 1349 cm^−1^ and G band centered at approximately 1584 cm^−1^. Notably, the wavenumbers of the D and G bands observed for the ERGO were highly similar to those of the D and G bands observed for the as-prepared GO. The D and G bands represented the defects and in-plane vibration of the sp^2^ carbon atoms, respectively. The ratio of the D-band and G-band feature peaks (I_D_/I_G_) was used to evaluate the difference in defect density between the as-prepared GO and ERGO flakes. When the as-prepared GO was converted to ERGO by increasing the reductive time under a negative voltage of −10 V, a slight red shift of the I_G_ peak was observed [46], and the I_D_/I_G_ ratio was progressively changed from 0.86 to 1.12. However, if a positive voltage of +10 V was provided for 4 h, the I_D_/I_G_ ratio decreased to 0.787. Thus, we speculate that GO oxidation was more obvious.

**Figure 4:**
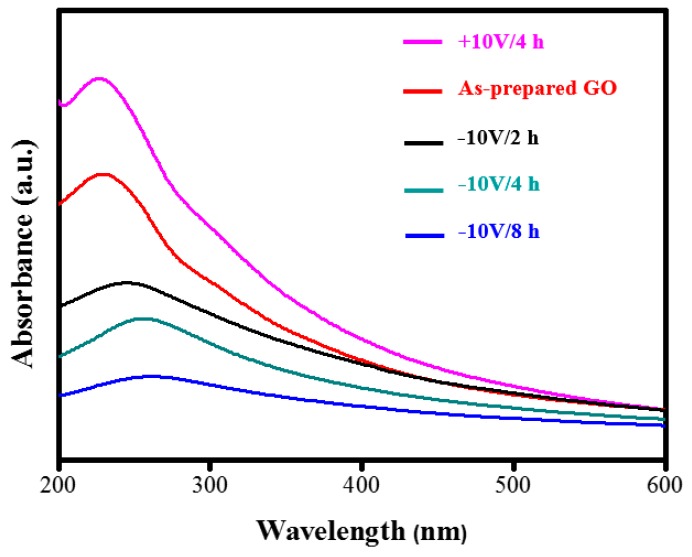
Comparison of UV-Vis spectroscopy among as-prepared GO and ERGO (+10 V/4 h, −10 V/2 h, −10 V/4 h, −10 V/8 h) suspension solutions.

Furthermore, the reduction of GO was also investigated by UV-Vis spectroscopy, as shown in Figure 4, using GO and ERGO suspension solutions. The change in the structures and morphologies of the GO and ERGO can be compared by such a UV-Vis spectroscopy. An obvious absorption peak at 228 nm was observed for as-prepared GO, corresponding to π→π* transitions of aromatic C=C bonds [47]. After electrochemical reduction using +10 V/4 h condition, the absorption peak of 228 nm is slightly blue-shifted to 225 nm. However, if the −10 Vcondition for 2 h treatment was used, the absorption of C=C bonds at 228 nm was obviously red-shifted to 250 nm. With increased time to 4 and 8 h, the absorption peak is continuously red-shifted to 261 nm and 263 nm, respectively. We also observed that the reduction was completed within 8 h, because the peak shifted no more when the reaction time was extended.The disappearance of the peak at 228 nm and the appearance of a new peak around 263 nm emerged in the UV spectra of the ERGO (−10 V/8 h), suggesting that sp^2^ carbon was restored and atoms were possibly rearranged within ERGO [48,49].

**Figure 5:**
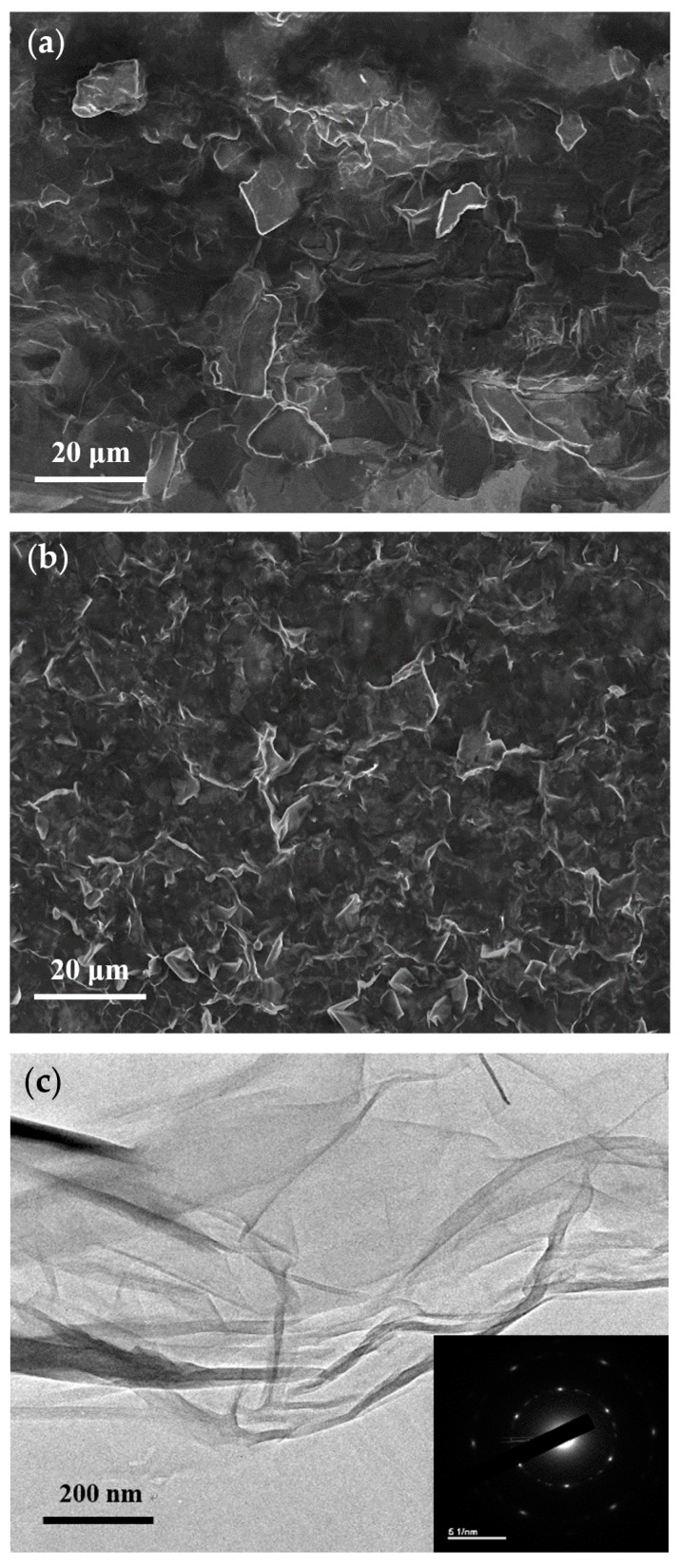
SEM morphologies of the (**a**) as-prepared GO and (**b**) ERGO treated with −10 V bias for 8 h. (**c**) High resolution TEM image and the selected area electron diffraction pattern (inset) from the sample (**b**).

Figure 5 depicts the SEM images of the samples, indicating an obvious morphological difference between the as-prepared GO and ERGO flakes treated with a −10 V bias for 8 h. The samples were dissolved in ethanol (99.5% *v/v*) to form a suspension, coated on silicon substrates by using a drop-casting technique, and then dried under the shade. As presented in Figure 5a, the GO flakes were more complete and had a larger area than the ERGO flakes. However, as presented in Figure 5b, the ERGO flakes exhibited a higher number of broken topographies. The reason for the higher number is that the ERGO flakes cyclically collided with each other and hit the rotating tool during the electrochemical reaction, thus causing the ERGO sheets to be torn into a distorted shape and shrink in area [17,50]. The ERGO material resembled a crumpled silk material, as displayed in the TEM images in Figure 5c [51]. From the selected area electron diffraction pattern presented in Figure 5c, a few-layer ERGO was presumed due to the obvious single-crystal structure.

**Figure 6:**
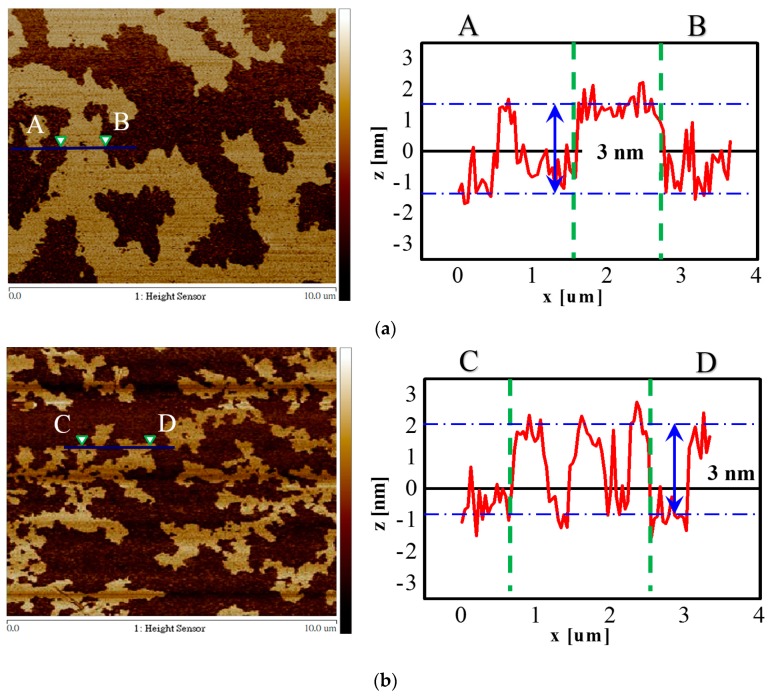
AFM images of (**a**) as-prepared GO and (**b**) ERGO treated with −10 V bias for 8 h and their height profiles on a SiO_2_/Si substrate.

AFM is currently the most widely used method for identifying the thickness of graphene layers. Figure 6 depicts the AFM images and the topographic profiles of the as-prepared GO and ERGO (−10 V for 8 h) samples. The AFM samples were prepared on silicon substrates by using drop casting and baked at a low temperature of 50 °C. The as-prepared GO flakes were distributed on the surface of silicon, and the thickness of the flakes was approximately 3 nm, as displayed in Figure 6a. The GO flakes were more complete and had a larger area (Figure 6a). However, the ERGO flakes exhibited a higher number of broken topographies and a smaller area but had a thickness of approximately 3 nm (Figure 6b). These results are similar to those in Figure 5a,b. We can reasonably infer that the rotor–stator tool played a crucial role in decreasing the size of the ERGO flakes during the electrochemical reduction.

**Figure 7:**
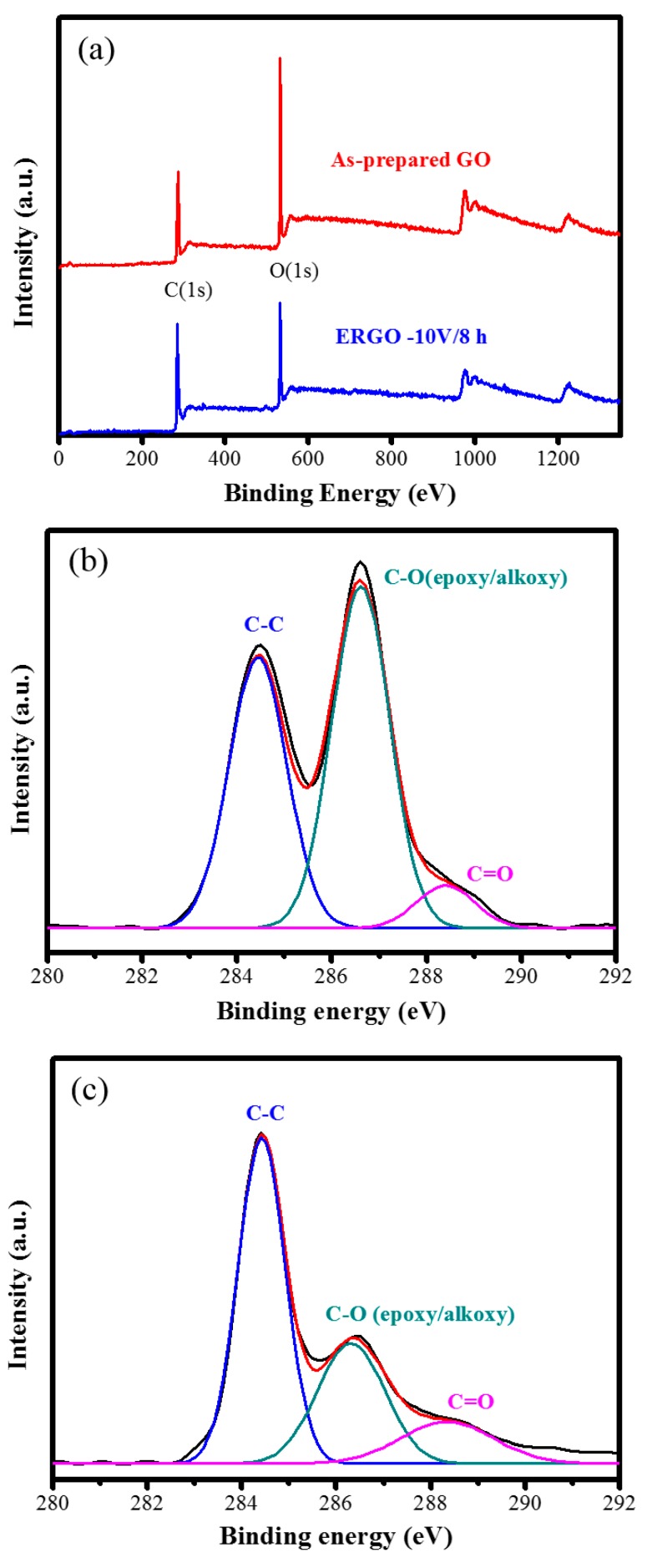
(**a**) XPS survey spectra, high-resolution C1s XPS spectra for (**b**) as-prepared GOand (**c**) ERGO treated with −10 V bias for 8 h.

To further determine the relative amount of carbon, oxygen, and functional groups present in graphene, XPS measurements were conducted on the as-prepared GO and ERGO samples. The XPS wide-region survey spectra of GO and ERGO are presented in Figure 7a. As demonstrated by the spectra, the O signal was higher than the C signal for the as-prepared GO sample, but the O signal decreased and became almost equal to the C signal for the ERGO sample. The high-resolution C1s XPS spectra of the as-prepared GO sample are presented in Figure 7b. Three different peaks centered at 284.5, 286.6, and 288.4 eV were observed, corresponding to the C–C group in aromatic rings, epoxy–alkoxy (C−O) group, and carboxylate (C=O) group, respectively [52]. For the spectra of the ERGO sample presented in Figure 7c, the intensities of all C1s peaks generated due to the binding of carbon to oxygen decreased; in particular, the peak of epoxy–alkoxy (C−O) group decreased drastically. This indicates that most of the oxygen-containing functional groups were removed after the electrochemical reduction. Table 1 summarizes the C–O ratio for three samples under various conditions (+10 V for 4 h, as-prepared GO, −10 V for 8 h). The carbon contents increased evidently for the sample prepared using +10 V for 4 h, the as-prepared GO, and the sample prepared using −10 V for 8 h, with the content levels being 62.2%, 66.9%, and 71.9%, respectively; however, the oxygen content decreased gradually, with the content levels being 37.8%, 33.1%, and 28.1%, respectively. Hence, the corresponding C–O ratios were calculated to be 1.64, 2.02, and 2.56, respectively.

**Figure 8:**
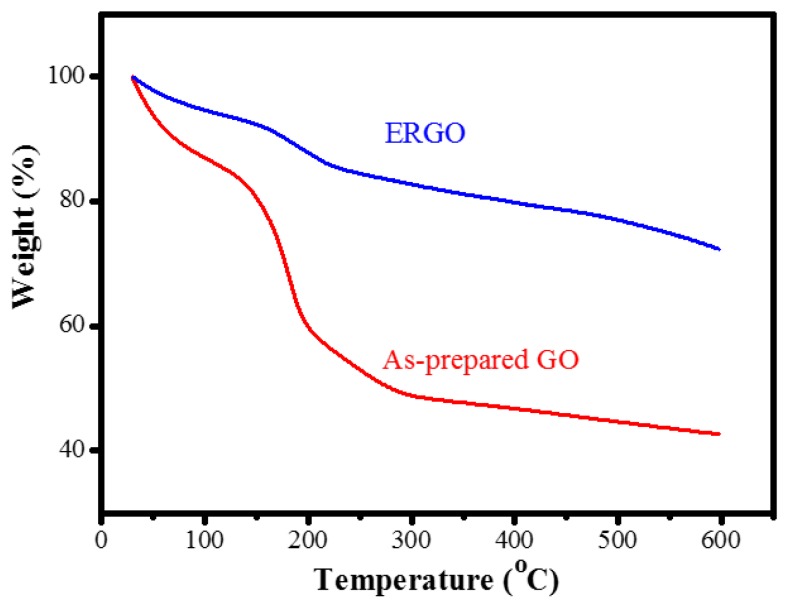
TGA plots of as-prepared GO and ERGO treated with −10 V bias for 8 h.

The thermal stability of the as-prepared GO and ERGO samples was also examined using the TGA method in a nitrogen atmosphere, as presented in Figure 8. The as-prepared GO sample began losing its mass, which was approximately 10 wt%, from 150 °C due to the removal of the moisture content. A major mass loss exceeding 50 wt% occurred at the temperature range of 150–600 °C due to the removal of labile oxygen-containing functional groups such as CO, CO_2_, and H_2_O vapors [26,53]. By contrast, the thermally labile oxygen functional groups were removed by electrochemical reduction. This caused the thermal stability of ERGO to increase, thus yielding a better thermal stability than that of the as-prepared GO. For the ERGO sample, approximately 5 wt% mass loss occurred in the first stage in the temperature range of 25–150 °C. In the second stage, 20 wt % loss occurred at 150–600 °C, which was much lower than that of the as-prepared GO. This finding indicates a decrease in the amount of oxygenated functional groups. This thereforedemonstrates that the removal of the thermally labile oxygen functional groups could increase the thermal stability of ERGO, indicating the success of the proposed electrochemical reduction technique.

**Figure 9:**
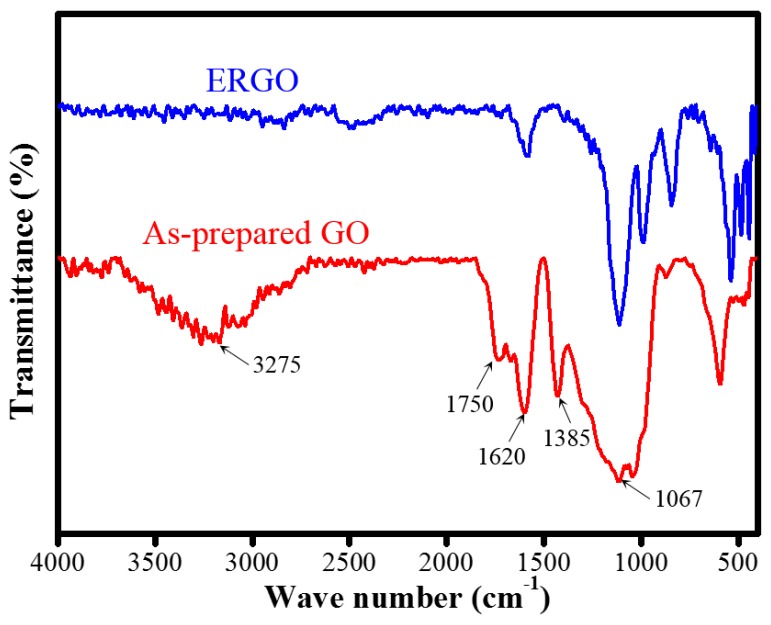
FTIR transmittance spectra ofas-prepared GO and ERGO treated with −10 V bias for 8 h.

FTIR spectroscopy was used to determine the degree of oxygen functional group removal for confirming the electrochemical reduction effect in the ERGO flakes. As shown in Figure 9, the spectrum of the as-prepared GO exhibited C=O (carboxylic and carbonyl at 1750 cm^−1^), C=C (unoxided graphite at 1620 cm^−1^), O−H (hydroxyl and deformation vibration of carboxyl at 3275 and 1385 cm^−1^), and C−O (epoxy or alkoxy at 1067 cm^−1^) [54,55]. The FTIR spectra of ERGO revealed a significantly attenuated peak band at 3275 cm^−1^ due to the disappearance of hydroxyl functionality. After the as-prepared GO was electrochemically reduced, the characteristic absorption bands of the oxide groups (O−H, C=O, and C=C) decreased drastically. This indicates the reduction of GO to ERGO and the removal of most of the oxygen-containing functional groups.

**Figure 10:**
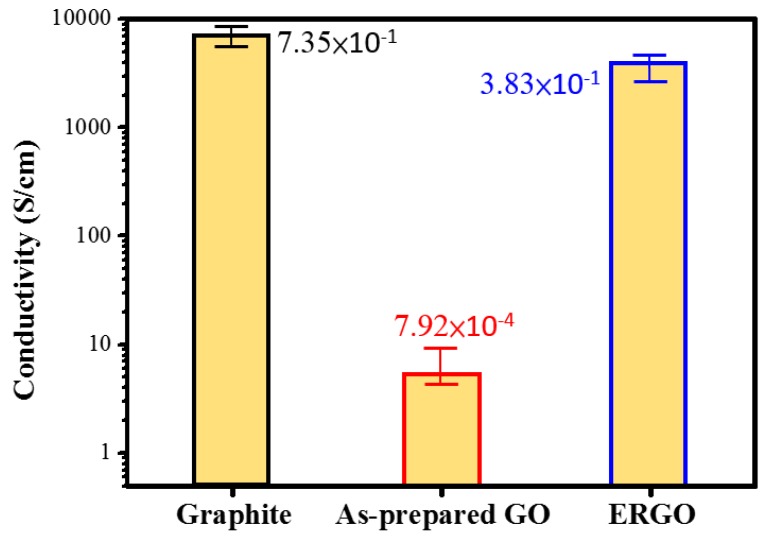
Electrical conductivity of thin films prepared from Graphite, as-prepared GO and ERGO treated with −10 V bias for 8 h.

Figure 10 shows the conductivity of the prepared graphite, GO flakes, and ERGO flakes measured using a four-point probe system. The error bars areobtained by measuring the conductivity of eight different specimens prepared from the same batched ERGO flakes.As shown in Figure 10, the conductivity of original graphite was 7.35 × 10^−1^ S·cm^−1^. The GO film exhibited an electrical conductivity of 7.92 × 10^−4^ S·cm^−1^, which was significantly restored to 3.83 × 10^−1^ S·cm^−1^ after the electrochemical reduction process executed using a −10 V bias for 8 h. Therefore, we conclude that the electrochemical reduction techniqueled to the restoration of the electrical properties of the as-prepared GO.The electrical conductivity value obtained for the electrochemical reduction technique is comparable to those obtained for other conventional chemical reduction techniques such as hydrazine (9.96 × 101 S·cm^−1^), NaBH_4_ (1.5 × 10^−2^ S·cm^−1^) [56], and thermal (2 × 10^−1^ S·cm^−1^) reduction [57]. The electrochemical reduction technique is expected to further increase the conductivity of GO films due to deoxygenation [58]; hence, the change in conductivity also provides evidence of GO reduction to ERGO through an electrochemical process. Finally, we have created a short table as Table 2, which gives the characteristic comparison between GO and ERGO to demonstrate that the electrochemical reduction technique proposed herein can obtain high-quality ERGO flakes.

## 4. Conclusions

This study proposes an environmentally friendly, energy-saving, and rapid technique for electrochemical reduction of as-prepared GO. This technique overcomes the drawback of conventional electrochemical reduction methods—which require the deposition of GO films on the working electrode—and has the potential for batch production. On the basis of the experimental results, the following conclusions can be drawn:

A technique in which GO flakes can be directly used as reductive materials was successfully developed. For this electrochemical technique, the GO films do not have to be coated on the cathode electrode in advance. Moreover, the technique has potential for large-scale and high-quality GO reduction.

After an electrochemical reaction executed using a −10 V bias for 8 h, the color of the suspension gradually changed from yellowish brown to black. Raman analysis results reveal that the ID/IG ratio increased from 0.86 to 1.12, and XPS measurement results reveal that the C/O ratio was increased from 2.02 to 2.56. These results demonstrate that the as-prepared GO flakes were reduced to engender ERGO flakes.

The ERGO flakes exhibited a higher amount of broken topographies than did the as-prepared GO flakes after electrochemical reduction. This is because the ERGO flakes cyclically collided with each other and hit the rotor–stator tool, which caused the ERGO flakes to have a larger specific surface area than that of the GO flakes.

According to the electrical conductivity analysis, the electrical conductivity of the ERGO film (3.83 × 10^−1^ S·cm^−1^) was considerably better than that of the GO film (7.92 × 10^−4^ S·cm^−1^) after electrochemical reduction executed using a −10 V bias for 8 h. This demonstrates that the proposed electrochemical reduction technique can be used to obtain ERGO flakes with high conductivity.

## Figures and Tables

**Table 1:** C/O ratio for the samples under various conditions.

Samples	C%	O%	C/O Ratio
ERGO +10 V/4 h	62.2	37.8	1.64
As-prepared GO	66.9	33.1	2.02
ERGO −10 V/8 h	71.9	28.1	2.56

**Table 2:** Characteristic comparison between GO and ERGO according to experimental results.

Measured Items	GO Sample	ERGO Sample (−10 V/8 h)
XRD 2θ peak (deg.)	10.2	NA
Raman I_D_/I_G_	0.86	1.12
UV-Vis absorbed wavelength (nm)	228	263
Ave. lateral dimension of flake (μm)	8	2
Ave. thickness of flake (nm)	3	3
XPS C/O (%)	2.02	2.56
TGA loss (wt%)	60	25
FTIR functional group	OH/C=O/C=C	Less
Electrical conductivity (S·cm^−1^)	7.92 × 10^−4^	3.83 × 10^−1^
